# Use of high-flow nasal cannula oxygen and risk factors for high-flow nasal cannula oxygen failure in critically-ill patients with COVID-19

**DOI:** 10.1186/s12931-022-02231-2

**Published:** 2022-12-03

**Authors:** Zakaria Ait Hamou, Nathan Levy, Julien Charpentier, Jean-Paul Mira, Matthieu Jamme, Mathieu Jozwiak

**Affiliations:** 1 Service de Médecine Intensive Réanimation, Hôpitaux Universitaires Paris Centre, Hôpital Cochin, Assistance Publique – Hôpitaux de Paris, 27 Rue du Faubourg Saint Jacques, 75014 Paris, France; 2 Université Paris Cité, Paris, France; 3 Service de Réanimation Médico-Chirurgicale, Hôpital Privé de l’Ouest Parisien, Ramsay Generale de Santé, 14 Rue Castiglione del Lago, 78190 Trappes, France; 4 INSERM U1018, Centre de Recherche en Épidémiologie et Santé des Populations (CESP), Equipe « Epidemiologie Clinique », Université Paris Saclay, 16 Avenue Paul Vaillant Couturier, 94800 Villejuif, France

**Keywords:** Acute respiratory distress syndrome, COVID-19, Intensive care unit, High-flow nasal cannula oxygen therapy, Prognosis, ROX index

## Abstract

**Background:**

High-flow nasal oxygen therapy (HFNC) may be an attractive first-line ventilatory support in COVID-19 patients. However, HNFC use for the management of COVID-19 patients and risk factors for HFNC failure remain to be determined.

**Methods:**

In this retrospective study, we included all consecutive COVID-19 patients admitted to our intensive care unit (ICU) in the first (Mars-May 2020) and second (August 2020- February 202) French pandemic waves. Patients with limitations for intubation were excluded. HFNC failure was defined as the need for intubation after ICU admission. The impact of HFNC use was analyzed in the whole cohort and after constructing a propensity score. Risk factors for HNFC failure were identified through a landmark time-dependent cause-specific Cox model. The ability of the 6-h ROX index to detect HFNC failure was assessed by generating receiver operating characteristic (ROC) curve.

**Results:**

200 patients were included: HFNC was used in 114(57%) patients, non-invasive ventilation in 25(12%) patients and 145(72%) patients were intubated with a median delay of 0 (0–2) days after ICU admission. Overall, 78(68%) patients had HFNC failure. Patients with HFNC failure had a higher ICU mortality rate (34 vs. 11%, p = 0.02) than those without. At landmark time of 48 and 72 h, SAPS-2 score, extent of CT-Scan abnormalities > 75% and HFNC duration (cause specific hazard ratio (CSH) = 0.11, 95% CI (0.04–0.28), per + 1 day, p < 0.001 at 48 h and CSH = 0.06, 95% CI (0.02–0.23), per + 1 day, p < 0.001 at 72 h) were associated with HFNC failure. The 6-h ROX index was lower in patients with HFNC failure but could not reliably predicted HFNC failure with an area under ROC curve of 0.65 (95% CI(0.52–0.78), p = 0.02). In the matched cohort, HFNC use was associated with a lower risk of intubation (CSH = 0.32, 95% CI (0.19–0.57), p < 0.001).

**Conclusions:**

In critically-ill COVID-19 patients, while HFNC use as first-line ventilatory support was associated with a lower risk of intubation, more than half of patients had HFNC failure. Risk factors for HFNC failure were SAPS-2 score and extent of CT-Scan abnormalities > 75%. The risk of HFNC failure could not be predicted by the 6-h ROX index but decreased after a 48-h HFNC duration.

**Supplementary Information:**

The online version contains supplementary material available at 10.1186/s12931-022-02231-2.

## Background

From December 2019, an emergent coronavirus Sars-CoV-2 is responsible for the Coronavirus disease 19 (Covid-19) worldwide pandemic [1]. Up two-thirds of hospitalized COVID-19 patients developed severe pneumonia, requiring their admission to intensive care unit (ICU) [2, 3].

Ventilatory support is a key point of the management of critically-ill COVID-19 patients [4, 5]. While high-flow nasal cannula oxygen therapy (HFNC) has been described as the most common ventilatory support in COVID-19 patients in China [6] and it has been suggested that HFNC use might be associated with lower intubation rate in the first pandemic wave [7–10], HFNC was used in only 19% of COVID-19 patients on ICU admission [11], because of the potential the initial recommendations, warning of the potential risk of aerosolization that could have increased the risk of contamination for healthcare workers [12, 13].

Thereafter, it has been shown that the risk of aerosolization with HFNC was similar to that with standard oxygen therapy [5, 14] and lower to that with non-invasive ventilation (NIV) [5], and HFNC has been suggested to be the first-line ventilatory support in COVID-19 patients with acute respiratory failure for the following pandemic waves [5]. In addition, risk factors for HFNC failure have been scarcely studied yet [6, 10, 15, 16], although up to more than 50% of HFNC failure have been reported in these patients [6, 8–11, 15, 16] and was associated with increased mortality [15]. In particular, although the ROX index, calculated as the ratio of pulse oximetry / inspired fraction of oxygen (SpO_2_/FiO_2_) over the respiratory rate [17, 18], has been shown to be a reliable predictor of HFNC failure or HFNC weaning [19] in non-COVID-19 patients, its ability to predict HFNC failure in patients with COVID-19 patients remains to be established [10, 16]. Thus, we aimed in this study to evaluate the use of HFNC as first-line ventilatory support in critically-ill patients with COVID-19.

## Methods

This retrospective study was conducted in a 24-bed ICU of a French university hospital. The study was approved by the Ethics committee of the Société de Réanimation de Langue Française (CE SRLF 20–72). Informed consent was waived but all patients or next of kin were informed about the study. The study complied with the Strengthening the Reporting of Observational Studies in Epidemiology (STROBE) statement guidelines.

We included all consecutive patients with severe COVID-19 pneumoniae admitted to our ICU in the first (March–May 2020) and second (August 2020- February 2021) French pandemic waves. All patients required at least 5 L/min of oxygen flow rate to achieve an arterial oxygen saturation ≥ 92%, delivered through a non-rebreather face mask. All patients had a positive real-time reverse transcriptase-polymerase chain reaction assay for SARS-CoV-2 in nasal swabs or pulmonary samples. Vaccination against COVID-19 had not yet started during the study period. All patients with a decision to withdraw life-sustaining therapy, including do-not-intubate orders, were excluded.

### Ventilatory management and respiratory measurements

In patients with HFNC (Optiflow®, Fisher and Paykel, Healthcare), oxygen was humidified by a heated humidifier and then applied continuously through large-bore binasal prongs. The gas flow rate was set at 60 L/min. The FiO_2_ was initially set at 100% and then adjusted to maintain SpO_2_ ≥ 92% and not higher than 96% [5]. HFNC was always used as first-line ventilatory support and was administered all day long until recovery or patients met criteria of intubation. The ROX index was calculated six hours after HFNC initiation, as it has been shown that patients with a 6-h ROX index ≥ 4.88 were less likely to be intubated, while patients with a 6-h ROX index < 3.47 were more likely to had HFNC failure [20].

NIV was performed with an ICU ventilator with NIV mode (CARESCAPE R860, GE Healthcare, Chicago, Il, USA or EVITA Infinity V500, Dräger, Antony, France). The initial pressure-support level was set at 8 cmH2O and then adjusted to obtain a tidal volume around 6 mL/kg of predicted body weight. The positive end-expiratory pressure level was set between 5 and 12 cmH2O and FiO_2_ was adjusted to maintain SpO_2_ ≥ 92% and not higher than 96% [5]. NIV was used either as first-line ventilatory support in patients not treated with HFNC, or as second-line ventilatory support after HNFC for oxygenation of patients before intubation.

The indication of intubation was left at the discretion of the attending physician based on the intubation criteria used in our ICU during the study period: (i) respiratory rate > 40 breaths/min, (ii) occurrence or lack of improvement of signs of high respiratory-muscle workload, (iii) occurrence of copious tracheal secretions, (iv) a SpO_2_ < 90% for more than five minutes without technical dysfunction and/or (v) respiratory acidosis with a pH < 7.35. After intubation, all patients were placed in 45-degree semi-recumbent position and mechanically ventilated in volume assist-controlled mode or in pressure regulated volume control mode. Tidal volume was set at 6 mL/kg of predicted body weight. Respiratory rate and the inspiratory/expiratory time ratio were adjusted to prevent respiratory acidosis and avoid dynamic hyperinflation. The positive end-expiratory pressure level was titrated to reach a maximum plateau pressure of 30 cmH2O with a maximum driving pressure of 15 cmH2O [21] and FiO_2_ was adjusted to maintain SpO_2_ ≥ 92% and not higher than 96% [5]. An airway humidification system was used in all patients.

Awake prone positioning sessions were performed in patients under HFNC or NIV [22]. Neuromuscular blocker agents and prone positioning sessions were used according to current recommendations for non-COVID-19 patients with acute respiratory distress syndrome [21].

### Data collection and endpoints

Demographic characteristics and comorbidities of patients, clinical, biological and radiological data, therapeutics as well as ICU clinical outcomes were collected and analyzed. Biological and radiological data were collected on ICU admission, at the initiation of HFNC and at the initiation of mechanical ventilation. The severity of CT-Scan abnormalities was assessed by the radiologist and divided into five categories according to the extent of ground-glass opacities and consolidations as a percentage of the total lung parenchyma: < 10%, 10–25%, 25–50%, 50–75 and > 75% [23].

The primary endpoint was the proportion of patients treated with HFNC as first-line ventilatory support. Secondary endpoints were the proportion of patients with HFNC failure, defined as the need for intubation after ICU admission, the risk factors for HFNC failure, the ability of the 6-h ROX index to predict HFNC failure, the intubation rate after ICU admission, the duration of invasive mechanical ventilation, the ICU length of stay and the mortality rate.

### Statistical analysis

Continuous variables were expressed as median (interquartile range) and categorical variables as numbers (percentages) and compared according pandemic waves by Kruskal–Wallis, Pearson’s Chi-square or Fisher exact test as appropriate. Patients treated with NIV as first-line ventilatory support were included in the “no HFNC group”, while patients treated with NIV as second-line ventilatory support after HFNC for oxygenation before intubation were included in the “HFNC group”.

Risk factors for HNFC failure were identified through a competing risk framework (*i.e.* Cox cause specific model), with ICU discharge alive or death in ICU without intubation as competing events and results were given as cause specific hazard ratio (CSH) with their 95% confidence interval (CI). All covariates related to ICU management (ventilatory management and treatments received during ICU stay) were assessed as time-dependent covariate. Because a minimum 48- and 72-h HFNC durations were considered for the different sensitive analyses, we performed a landmark time-dependent cause-specific Cox proportional hazard model. Landmarking is a common method recommended for the analysis of time-dependent covariates in time-to-event data to take into account immortal bias induced by a minimum exposure of covariates [24, 25]. All variables included in the Cox cause specific model were either variables selected a priori based on published and experts’ knowledge of known risk factors for HFNC failure or variables with a p-value < 0.20 in univariate time-dependent analysis: age, SAPS-2 score, body mass index, intensity of thrombophylaxis, extent of CT-Scan abnormalities > 75%, lymphocytes count, fibrinogen level, D-Dimer level, interleukin-6 level, corticosteroids administration, immunomodulatory treatments administration, antiviral drugs administration, antibiotherapy administration, 6-h ROX index, HFNC duration and delay from onset of symptoms to HFNC initiation. To assess the ability of the 6-h ROX index to detect HFNC failure, receiver operating characteristic curve (with 95% CI) was generated and the best threshold value was determined so as to maximize the Youden Index (specificity + sensitivity – 1).

The impact of HFNC use was analyzed in the whole cohort and after constructing a propensity score using logistic regression, based on the following variables: age, gender, pandemic wave, Charlson score, body mass index, extent of CT-Scan abnormalities, use of awake prone positioning, corticosteroids administration, antiviral drugs administration, thrombophylaxis administration, intensity of thrombophylaxis, antibiotherapy administration, and biological variables (lymphocytes count, fibrinogen and D-Dimers) on ICU admission. Subjects were 1:1 matched without replacement by the estimated propensity score using nearest neighbor matching with a caliper of 0.2 standard differences of the logit of the propensity score. Standardized differences were determined to ascertain balance between the propensity-matched groups. Cox cause-specific analysis in matched cohort was performed to assess the relationship between HFNC use and the risk of intubation.

All analyses were carried out using R 3.1.1 (R foundation for Statistical Computing Vienna, Austria). Missing values of covariates in the multivariable models were handled by multiple imputations with chained equations, based on M = 30 imputed complete datasets, with an estimated hazard ratio based on the average value of the regression coefficients [26]. All tests were two-sided, with p values of 0.05 or less denoting statistical significance.

## Results

### Study population

**Fig. 1:**
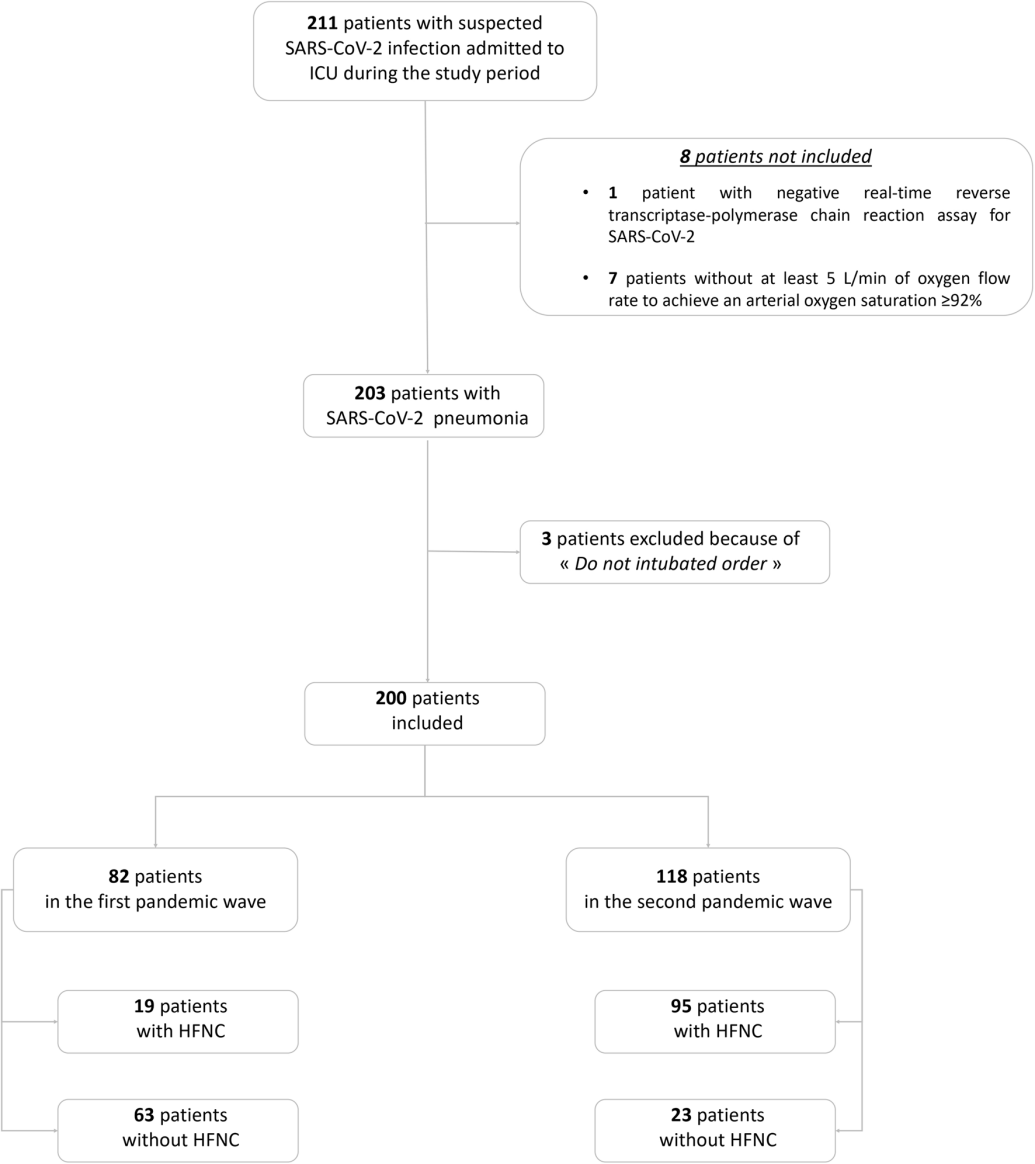
Flowchart of the study. *ICU* Intensive Care Unit, *HFNC* high-flow nasal cannula oxygen therapy

In the two pandemic waves, 211 patients with suspected SARS-CoV-2 infection were admitted to our ICU and 200 patients were finally included: 82 in the first wave and 118 in the second wave (Fig. 1). Among the 200 patients, 70% were men, with a median age of 64 (55–73) years old and a median SAPS-2 score of 46 (34–70), all patients had at least one cardiovascular comorbidity, 41 (20%) were immunocompromised, 116 (58%) received corticosteroids and 75 (37%) were intubated within the first 24 h after ICU admission. The ICU mortality rate was 28% (Table 1 and Additional file 1: Table S1). Characteristics and outcomes of patients according to the pandemic wave are summarized in Additional file 1: Table S2.

**Table 1:** Patient characteristics according to the use of high-flow nasal cannula oxygen therapy

Variables	No HFNC (n = 86)	HFNC (n = 114)	p value
Clinical characteristics			
Age (years)	62 (51–70)	68 (59–74)	0.003
SAPS-2 score	65 (42–77)	41 (31–60)	< 0.001
SOFA score on ICU admission	9 (4–12)	5 (4–7)	< 0.001
Male gender, n (%)	56 (65)	85 (74)	0.20
Body mass index (kg/m^2^)	27 (26–32)	28 (25–30)	0.65
Obesity, n(%)	29 (34)	31 (28)	0.40
Arterial hypertension, n (%)	28 (32)	68 (60)	0.12
Diabetes mellitus, n (%)	25 (30)	40 (34)	0.54
Coronary artery disease, n (%)	9 (10)	19 (17)	0.30
Stroke, n (%)	7 (8)	5 (4)	0.42
Chronic respiratory disease, n (%)	10 (12)	21 (18)	0.95
Chronic kidney disease, n (%)	11 (13)	16 (14)	0.96
Cirrhosis, n (%)	1 (1)	2 (2)	1.00
Neoplasia, n (%)	16 (19)	22 (19)	1.00
Blood type, n (%)*			0.72
A	35 (42)	45 (41)	
B	12 (14)	16 (14)	
AB	7 (8)	5 (5)	
O	30 (36)+ 36)	44 (40)	
Second pandemic wave, n (%)	23 (27)	95 (83)	< 0.001
Nosocomial COVID-19, n (%)	7 (8)	9 (8)	1.00
CT-Scan, n (%)	68 (79)	102 (89)	0.06
CT-Scan abnormalities (%)			0.06
< 10%	3 (5)	3 (3)	
10–25%	12 (17)	23 (23)	
25–50%	22 (33)	39 (38)	
50–75%	25 (36)	30 (29)	
> 75%	6 (9)	7 (7)	
Norepinephrine, n (%)	54 (63)	68 (60)	0.76
Renal replacement therapy, n (%)	22 (26)	22 (13)	0.37
Biological variables at ICU admission			
Lymphocytes (× 10^9^/L)	0.77 (0.55–1.18)	0.72 (0.48–1.11)	0.27
Fibrinogen (g/L)	5.7 (4.8–7.3)	6.0 (5.0–7.0)	0.69
D-Dimers (µg/L)	2308 (1253–6831)	1180 (642–2227)	< 0.001
Protein C reactive (mg/L)	136 (43–470)	130 (72–200)	0.65
Procalcitonin (ng/L)	0.48 (0.17–1.65)	0.28 (0.13–0.72)	0.04
Ferritin (ng/mL)	948 (409–3576)	1215 (563–4106)	0.87
Interleukin-6 (pg/mL)	113 (43–471)	54 (17–130)	< 0.001
Troponin (ng/L)	25 (11–155)	17 (10–57)	0.59

n = 200. Data are expressed as median (interquartile range) or counts (percentages)

*HFNC* high-flow nasal cannula oxygen therapy, *ICU* intensive care unit, *SAPS* simplified acute physiology score, *SOFA* sepsis-related organ failure assessment

*Data are available for 194 patients: 84 patients in the “no HFNC” group and 110 patients in the “HFNC” group

### HFNC use

Overall, HFNC was used in 114 (57%) patients, NIV in 25 (12%) patients, 145 (72%) patients were intubated with a median delay of 0 (0–2) days after ICU admission and 14 (7%) patients required venovenous extracorporeal membrane oxygenation.

Patients with HFNC were older (68 (59–74) vs. 62 (51–70) years old, p = 0.003) and less severe (41 (31–60) vs. 65 (42–77) SAPS-2, p < 0.001) than those without HFNC. The other clinical characteristics were similar between both groups of patients (Table 1). The delay from the onset of symptoms to ICU admission was 8 (6–11) days and from the onset of symptoms to HFNC initiation 8 (5–11) days. The duration of HFNC was 3 (1–6) days.

HFNC use was more frequent (80 vs. 23%, p < 0.001) and HFNC duration was longer (3 (1–6) vs. 0 (0–1) days, p < 0.001) in the second than in the first pandemic wave, while the delay of HFNC initiation was not different in both pandemic waves (Additional file 1: Table S2, Fig. 1). Ventilatory management according to the pandemic wave is summarized in Additional file 1: Table S2.

### Risk factors for HFNC failure

Among the 114 (57%) patients with HFNC, 78 (68%) had HFNC failure and 31 (27%) died. Patients with HFNC failure had a higher SAPS-2 score, a higher SOFA score on ICU admission than patients without HFNC failure (Table 2). Patients with HFNC failure had a higher ICU mortality rate (34 vs. 11%, p = 0.02) and a longer ICU length of stay (16 (7–33) *vs.* 5 (3–8) days, p < 0.001) than those without HFNC failure (Table 2).

**Table 2:** Patient characteristics, management and outcomes according to high-flow nasal cannula oxygen therapy failure

Variables	No HFNC failure (n = 36)	HFNC failure (n = 78)	p value
Clinical characteristics			
Age (years)	65 (56–74)	69 (61–74)	0.41
SAPS-2 score	31 (24–39)	48 (37–70)	< 0.001
SOFA score on ICU admission	4 (3–5)	5 (4–9)	0.01
Male gender, n (%)	26 (72)	59 (76)	0.87
Body mass index (kg/m^2^)	28 (26–31)	28 (25–30)	0.53
Obesity, n(%)	10 (28)	21 (27)	1.00
Arterial hypertension, n (%)	19 (53)	49 (63)	0.42
Diabetes mellitus, n (%)	11 (31)	28 (36)	0.73
Coronary artery disease, n (%)	6 (17)	13 (17)	1.00
Stroke, n (%)	2 (6)	3 (4)	1.00
Chronic respiratory disease, n (%)	2 (6)	15 (19)	0.10
Chronic kidney disease, n (%)	5 (14)	11 (14)	1.00
Cirrhosis, n (%)	0 (0)	2 (3)	0.84
Neoplasia, n (%)	5 (14)	17 (22)	0.46
Blood type, n (%)*			0.83
A	12 (37)	33 (42)	
B	6 (19)	10 (13)	
AB	1 (3)	4 (5)	
O	13 (41)	31 (40)	
Second pandemic wave, n (%)	31 (86)	64 (82)	0.41
Nosocomial COVID-19, n (%)	2 (5)	7 (9)	0.80
CT-Scan, n (%)	34 (94)	71 (91)	0.80
CT-Scan abnormalities (%)			0.34
< 10%	0 (0)	2 (3)	
10–25%	8 (24)	18 (26)	
25–50%	16 (47)	25 (35)	
50–75%	10 (29)	20 (28)	
> 75%	0 (0)	6 (8)	
Norepinephrine, n (%)	0 (0)	68 (87)	< 0.001
Renal replacement therapy, n (%)	2 (6)	20 (26)	0.01
Biological variables at ICU admission			
Lymphocytes (× 10^9^/L)	0.77 (0.58–1.02)	0.61 (0.40–1.06)	0.11
Fibrinogen (g/L)	6.52 (5.54–7.12)	5.59 (5.05–7.00)	0.13
D-Dimers (µg/L)	1094 (634–1815)	1055 (642–1802)	0.77
Protein C reactive (mg/L)	128 (61–200)	136 (94–180)	0.40
Procalcitonin (ng/L)	0.20 (0.10–0.55)	0.31 (0.16–0.62)	0.23
Ferritin (ng/mL)	1066 (598–1259)	1090 (705–2053)	0.63
Interleukin-6 (pg/mL)	32 (19–42)	69 (21–197)	0.07
Troponin (ng/L)	15 (11–81)	23.5 (14–42)	0.03
Treatments at ICU admission			
Corticosteroids, n (%)	31 (86)	63 (81)	0.67
Immunomodulatory treatments, n (%)	2 (6)	4 (5)	1.00
Antiviral drugs, n (%)	2 (6)	7 (9)	0.79
Low-dose thrombophylaxis, n (%)	4 (11)	13 (17)	0.62
Enhanced intermediate-dose thrombophylaxis, n (%)	31 (86)	52 (67)	0.05
Curative anticoagulation, n (%)	1 (3)	14 (18)	0.05
Antibiotherapy, n (%)	15 (42)	43 (55)	0.26
Ventilatory management			
6-h ROX index	1.88 (1.46–2.10)	1.56 (1.33–1.83)	0.02
Non-invasive ventilation, n (%)	6 (17)	14 (18)	1.00
Intubation, n (%)	0 (0)	78 (100)	< 0.001
Neuromuscular blocker agents, n (%)	0 (0)	74 (95)	< 0.001
Prone positioning, n (%)	0 (0)	61 (78)	< 0.001
Number of prone positioning sessions	0 (0–0)	3 (2–5)	NA
Awake prone positioning, n (%)	17 (47)	30 (39)	0.50
Venovenous ECMO, n (%)	0 (0)	7 (9)	0.15
Nitric oxide, n (%)	0 (0)	14 (18)	0.02
Delays and outcomes			
From onset of symptoms to ICU admission (days)	9 (7–11)	7 (5–10)	0.03
From onset of symptoms to HFNC initiation (days)	9 (7–11)	7 (5–10)	0.01
From ICU admission to HFNC initiation (days)	0 (0–1)	0 (0–1)	0.93
From ICU admission to intubation (days)	0 (0–0)	2 (1–5)	< 0.001
Duration of HFNC (days)	5 (3–9)	2 (2–6)	< 0.001
Duration of invasive mechanical ventilation (days)	0 (0–0)	6 (0–16)	< 0.001
Tracheostomy, n (%)	0 (0)	16 (20)	0.01
Ventilator-associated pneumonia, n (%)	0 (0)	63 (81)	< 0.001
Pulmonary embolism, n (%)	0 (0)	5 (6)	0.29
Pneumothorax, n (%)	0 (0)	4 (5)	0.40
ICU length of stay (days)	5 (3–8)	16 (7–33)	< 0.001
ICU mortality (n,%)	4 (11)	27 (34)	0.02
In-hospital mortality, n (%)	6 (17)	28 (36)	0.06

n = 114. Data are expressed as median (interquartile range) or counts (percentages)

*ECMO* extracorporeal membrane oxygenation, *HFNC* high-flow nasal cannula oxygen therapy, *ICU* intensive care unit, *SAPS* simplified acute physiology score, *SOFA* sepsis-related organ failure assessment

*Data are available for 110 patients: 32 patients in the “no HFNC failure” group and 78 patients in the “HFNC failure” group

At landmark time of 48 h, SAPS-2 score (CSH = 1.55, 95% CI (1.26–1.92) per + 10 points, p < 0.001), extent of CT-Scan abnormalities > 75% (CSH = 28.94, 95% CI (3.14–266.02), p = 0.003) and HFNC duration (CSH = 0.11, 95% CI (0.04–0.28), per + 1 day, p < 0.001) were associated with HFNC failure. At landmark time of 72 h, SAPS-2 score (CSH = 1.60, 95% CI (1.21–2.13) per + 10 points, p < 0.001), extent of CT-Scan abnormalities > 75% (CSH = 10.48, 95% CI (1.92–118.22), p = 0.03) and HFNC duration (CSH = 0.06, 95% CI (0.02–0.23), per + 1 day, p < 0.001) were associated with HFNC failure (Table 3).

**Table 3:** Risk factors for high-flow nasal cannula oxygen therapy failure

Variables	Landmark 48 h risk set (n = 72)			Landmark 72 h risk set (n = 53)		
	CSH	95%CI	p value	CSH	95%CI	p value
SAPS-2 score (+ 10 points)	1.55	1.26–1.92	< 0.001	1.60	1.21–2.13	< 0.001
CT-Scan abnormalities > 75%	28.94	3.14–266.02	0.003	10.48	1.92–118.22	0.03
6-h ROX index (+ 1 point)	0.92	0.44–2.28	0.85	0.98	0.47–3.76	0.58
HFNC duration from landmark (+ 1 day)*	0.11	0.04–0.28	< 0.001	0.06	0.02–0.23	< 0.001

Covariables included in the landmark time-dependent cause-specific Cox proportional hazard model before stepwise covariate selection: Age; SAPS-2 score; Body mass index; Intensity of thrombophylaxis; extent of CT-Scan abnormalities > 75%; Lymphocytes count; Fibrinogen level; D-Dimer level; Interleukin-6 level; Corticosteroids administration; Immunomodulatory treatments administration; Antiviral drugs administration; Antibiotherapy administration; 6-h ROX index; HFNC duration and delay from onset of symptoms to HFNC initiation

*CI* confidence interval, *CSH* cause specific hazard ratio, *HFNC* high-flow nasal cannula oxygen therapy, *SAPS* simplified acute physiology score

*Time-dependent covariate: 38 patients had HFNC failure for the 48 h analysis and 28 patients had HFNC failure for the 72 h analysis

**Fig. 2:**
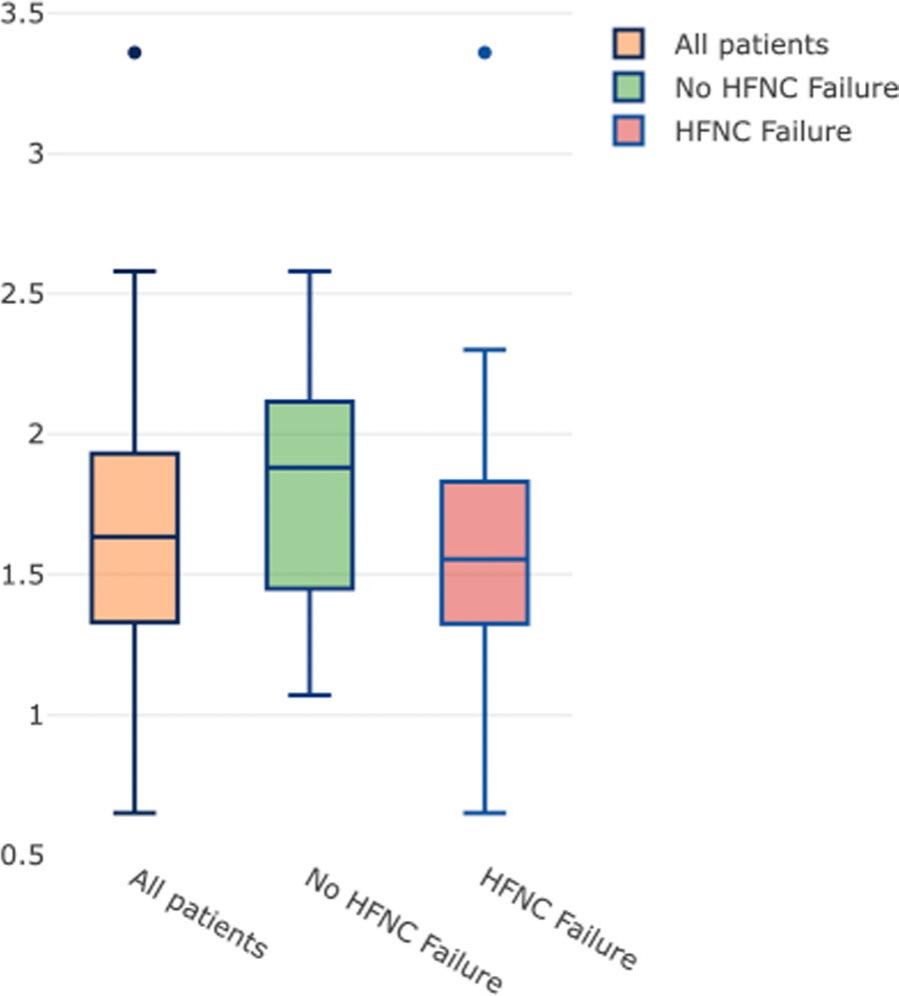
ROX index calculated six hours after high-flow nasal cannula oxygen therapy (HFNC) initiation (6-h ROX index) in all patients with HFNC (n = 114), in patients with HFNC failure (n = 78) and in patients without HFNC failure (n = 36). The boxes show the 25th and 75th percentiles, the line in the box the median, and the whiskers the minimum and maximum values. Circles represent outliers

**Fig. 3:**
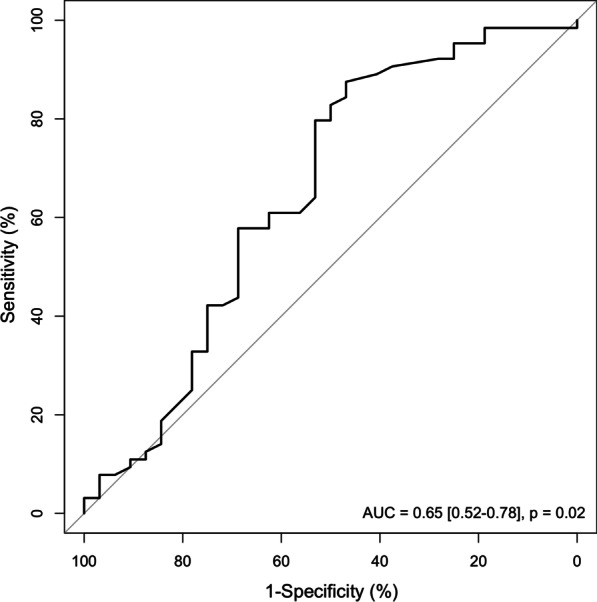
Receiver operating characteristics curve for the ability of the ROX index calculated six hours after high-flow nasal cannula oxygen therapy (HFNC) initiation (6-h ROX index) to predict HFNC failure (n = 114). *AUC* area under the curve, expressed as mean [95% confidence interval]

The 6-h ROX index was lower in patients with HFNC failure (1.56 (1.33–1.83) *vs.* 1.88 (1.46–2.10), p = 0.02) than in those without HFNC failure. All patients had a 6-h ROX index < 3.47 (Table 2, Fig. 2). HFNC failure was predicted by a 6-h ROX index of 1.96 with a sensitivity of 47% (95% CI: 32–62%) and a specificity of 87% (95%CI: 80–94%) (Fig. 3). A 6-h ROX index < 1.10 predicted HFNC failure with 100% (95% CI: 98–100%) specificity.

### Impact of HFNC use

The intubation rate was not different (68 vs. 78%, p = 0.18) between patients with and without HFNC. Patients with HFNC were intubated later after ICU admission (2 (1–4) vs. 0 (0–0) days, p < 0.001) and had shorter duration of mechanical ventilation (16 (7–33) vs. 24 (13–36) days, p = 0.007) than those without HFNC. The ICU mortality rate and the ICU length of stay were not different between both groups of patients (Additional file 1: Table S1).

**Fig. 4:**
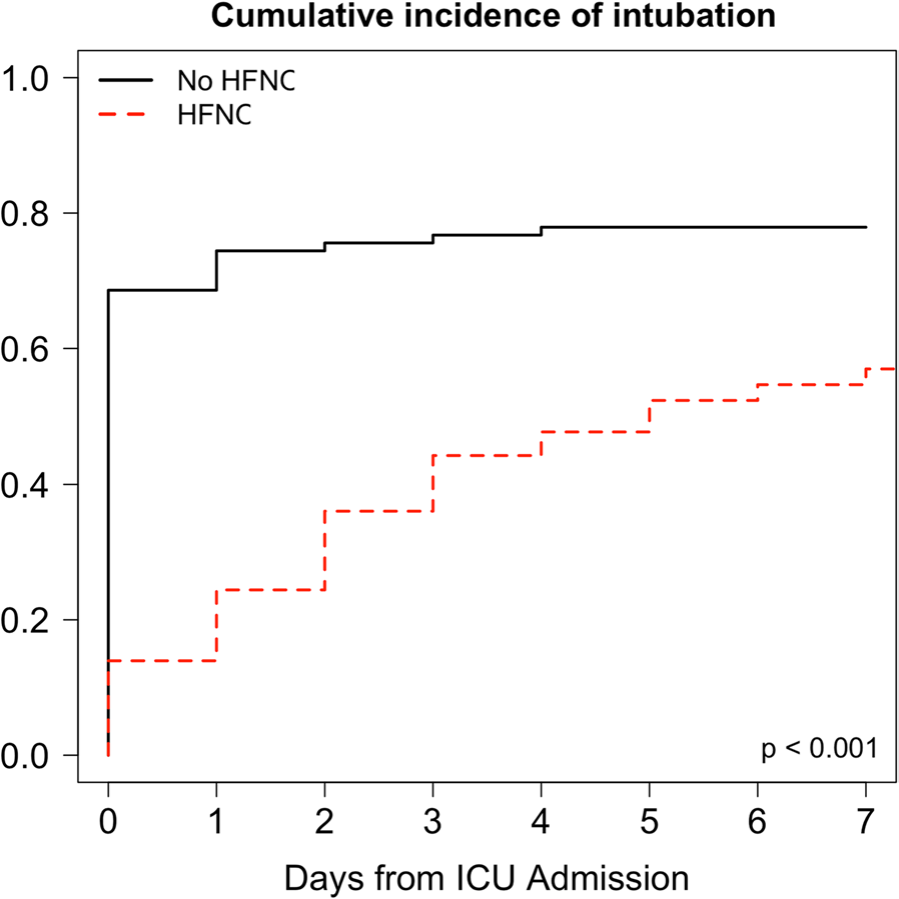
Cumulative incidence of intubation after admission in intensive care unit (ICU) in the matched cohort of patients after propensity score without (solid black line, n = 35) and with (dotted red line, n = 35) high-flow nasal cannula oxygen therapy (HFNC)

Similar results were found in the matched cohort of 70 patients after propensity score (Table 4). In the matched cohort, HFNC use was associated with a lower risk of intubation (CSH = 0.32, 95% CI (0.19–0.57), p < 0.001) (Fig. 4).

**Table 4:** Patient characteristics, management and outcomes in the matched cohort according to the use of high-flow nasal cannula oxygen therapy

Variables	No HFNC (n = 35)	HFNC (n = 35)	p value
Clinical characteristics			
Age (years)	64 (53–75)	65 (52–70)	0.60
SAPS2 score	48 (36–78)	42 (31–57)	0.16
SOFA score at ICU admission	5 (3–10)	6 (4–10)	0.68
Male gender, n (%)	22 (63)	22 (63)	1.00
Body mass index (kg/m^2^)	27 (26–31)	28 (24–32)	0.99
Obesity, n (%)	11 (31)	13 (37)	0.80
Arterial hypertension, n (%)	17 (49)	19 (54)	0.81
Diabetes mellitus, n (%)	13 (37)	11 (31)	0.80
Coronary artery disease, n (%)	6 (17)	7 (20)	1.00
Stroke, n (%)	4 (11)	3 (9)	1.00
Chronic respiratory disease, n (%)	5 (14)	7 (20)	0.75
Chronic kidney disease, n (%)	1 (3)	1 (3)	1.00
Cirrhosis, n (%)	1 (3)	1 (3)	1.00
Neoplasia, n (%)	4 (11)	3 (8)	1.00
Blood type, n (%)			0.16
A	16 (45)	20 (57)	
B	8 (23)	3 (9)	
AB	2 (6)	0 (0)	
O	9 (26)	12 (34)	
Second pandemic wave, n (%)	18 (51)	20 (57)	0.81
Nosocomial COVID-19, n (%)	32 (91)	33 (94)	1.00
CT-Scan, n (%)	30 (86)	32 (91)	0.71
CT-Scan abnormalities (%)			0.36
< 10%	1 (3)	0 (0)	
10–25%	9 (30)	5 (17)	
25–50%	7 (23)	12 (37)	
50–75%	11 (37)	12 (37)	
> 75%	2 (7)	3 (9)	
Norepinephrine, n (%)	20 (57)	19 (54)	1.00
Renal replacement therapy, n (%)	10 (29)	6 (17)	0.39
Biological variables at ICU admission			
Lymphocytes (× 10^9^/L)	0.92 (0.44–1.42)	0.86 (0.55–1.12)	0.30
Fibrinogen (g/L)	5.88 (5.05–7.88)	5.99 (5.49–7.11)	0.99
D-Dimers (µg/L)	2231 (737–3666)	1187 (858–4545)	0.62
Protein C reactive (mg/L)	150 (87–193)	165 (100–258)	0.48
Procalcitonin (ng/L)	0.49 (0.20–1.25)	0.45 (0.12–1.01)	0.51
Ferritin (ng/mL)	1137 (635–3238)	808 (450–1491)	0.14
Interleukin-6 (pg/mL)	175 (63–422)	129 (63–336)	0.64
Troponin (ng/L)	15 (11–73)	30 (14–49)	0.46
Treatments at ICU admission			
Corticosteroids, n (%)	18 (51)	21 (60)	0.63
Immunomodulatory treatments, n (%)	0 (0)	5 (14)	0.07
Antiviral drugs, n (%)	8 (23)	7 (20)	1.00
Low-dose thrombophylaxis, n (%)	13 (37)	9 (26)	0.44
Enhanced intermediate-dose thrombophylaxis, n (%)	17 (49)	20 (57)	0.63
Curative anticoagulation, n (%)	5 (14)	6 (17)	1.00
Antibiotherapy, n (%)	20 (57)	17 (49)	0.63
Ventilatory management			
Non-invasive ventilation, n (%)	4 (11)	6 (17)	0.73
Intubation, n (%)	25 (71)	24 (69)	1.00
Neuromuscular blocker agents, n (%)*	22(88)	22 (92)	1.00
Prone positioning, n (%)*	11 (44)	15 (62)	0.25
Number of prone positioning sessions	0 (0–2)	0 (0–3)	0.52
Awake prone positioning, n (%)	1 (3)	3 (9)	0.61
Venovenous ECMO, n (%)*	0 (0)	3 (12)	0.11
Nitric oxide, n (%)*	0 (0)	3 (12)	0.11
Delays and outcomes			
From onset of symptoms to ICU admission (days)	7 (6–10)	9 (7–12)	0.13
From ICU admission to intubation (days)	0 (0–0)	2 (1–5)	< 0.001
Duration of invasive mechanical ventilation (days)	13 (0–27)	6 (0–16)	0.003
Tracheostomy, n (%)*	3 (12)	3 (12)	1.00
Ventilator-associated pneumonia, n (%)*	18 (72)	20 (83)	0.49
Pulmonary embolism, n (%)	1 (3)	2 (6)	1.00
Pneumothorax, n (%)	1 (3)	1 (3)	1.00
ICU length of stay (days)	14 (2–28)	10 (5–26)	0.98
ICU mortality (n,%)	8 (23)	10 (29)	0.78
In-hospital mortality, n (%)	10 (29)	11 (31)	1.00

n = 70. Data are expressed as median (interquartile range) or counts (percentages)

*ECMO* extracorporeal membrane oxygenation, *HFNC* high-flow nasal cannula oxygen therapy; *ICU* intensive care unit, *SAPS* simplified acute physiology score, *SOFA* sepsis-related organ failure assessment *In patients who were intubated: n = 25 in the “No HFNC” group and n = 24 in the “HFNC” group

## Discussion

Although the place of non-invasive respiratory support in critically-ill COVID-19 patients is still debated because of the potential issues of barotrauma and/or patient self-inflicted lung injury [27–30], non-invasive respiratory support may become increasingly important in the ventilatory management of these patients. We found that HFNC was used as first-line ventilatory support in 57% of patients and was associated with a lower risk of intubation. However, 68% of patients had HFNC failure and patients with HFNC failure had increased mortality. In multivariate analysis, SAPS-2 score and extent of CT-Scan abnormalities > 75% were associated with a higher risk of HFNC failure, while a minimum 48-h and 72-h HFNC duration was associated with a lower risk of HFNC failure. The 6-h ROX index could not reliably predict HFNC failure.

In our cohort, HFNC was used as first-line ventilatory support in 57% of patients with COVID-19, and HFNC use increased between the first two pandemic waves, reaching 80% of patients in the second wave, which is consistent with existing literature showing increased use of HFNC across pandemic waves [31]. This increased HFNC use is probably related to the fact that the risk of aerosolization with this ventilatory support is lower than initially suspected [5, 14]. Although HFNC use delayed intubation, the duration of invasive mechanical ventilation was shorter in patients with HFNC and the ICU mortality rate was not different between patients with and without HFNC. In addition, HFNC use was associated with a lower risk of intubation, as previously demonstrated in the most severe non-COVID-19 patients with hypoxemic acute respiratory failure compared to those treated with NIV or standard oxygen therapy [32, 33]. Our results are consistent with those of previous observational studies [7–10, 31, 34] and those of a recent randomized trial comparing HFNC and conventional oxygen therapy [35] in patients with COVID-19. Nevertheless, it must be noted that other randomized trials found conflicting results regarding the effects of using HFNC as first-line ventilatory support in COVID-19 patients with moderate to severe acute respiratory failure on intubation rate [36, 37] and further studies are needed to address this issue.

However, 68% of patients had HFNC failure, defined as the need for intubation, which is higher than the range of 32 to 57% of HFNC failure reported in critically-ill COVID-19 patients [6, 8–10, 15, 34] and to the overall intubation rate of 37% in patients with COVID-19 treated with non-invasive respiratory support (HFNC or NIV) found in a systematic review of the literature [38]. This discrepancy may be explained by the heterogeneity of the definition of HFNC failure used between the different studies and/or the different severity of patients included in the different studies. In addition, this discrepancy should be considered with caution as the intubation rate depends on ICU admission criteria and the ventilatory management of patients, which may differ between ICUs and countries due to potentially different local organization of care in the absence of strong international recommendations. Patients with HFNC failure had a higher ICU mortality rate and a longer ICU length of stay than those without. The 34% ICU mortality rate we found was significantly lower than the 65% reported by Xia and colleagues [15]. Such difference may be explained by the fact that HFNC failure was defined as upgrading respiratory support to positive pressure ventilation or death after HFNC use [15] and not as the need for intubation and/or by the fact that the median delay to intubation in patients with HFNC failure was 1.5 days longer than in our study [15].

These worse outcomes highlight the importance for intensivists of having reliable early predictors of HFNC failure in patients with COVID-19. To date, only SAPS-2 score [10], male gender and lower oxygenation before HFNC initiation [6, 15] have been reported to be associated with a higher risk of intubation in these patients. In our cohort, SAPS-2 score only was a risk factor of HFNC failure, confirming previous result from Bonnet and colleagues [10]. To our knowledge, our study is the first to report that a minimum 48-h and 72-h HFNC duration was associated with a lower risk of HFNC failure. This suggests that HFNC failure probably occurs early in most patients, as illustrated by the median delay from ICU admission and intubation of 2 (1–5) days. Furthermore, these results may suggest that after a 48-h HFNC duration, intensivists may be partly reassured about the risk of HFNC failure and may consider continuing to manage these patients with this oxygenation technique, bearing in mind that many other confounding factors may be involved in the favorable or unfavorable evolution of patients with COVID-19.

Previous studies have also shown that the ROX index was associated with the risk of intubation in COVID-19 patients [10, 16]. Here, we found that patients with HFNC failure had a lower 6-h ROX index than those without HFNC failure, as previously shown in COVID-19 patients [15], but that the reliability of the 6-h ROX index in predicting HFNC failure was low, consistent with previous findings in immunocompromised patients with hypoxemic acute respiratory failure [39]. Some reasons may explain these discrepancies between our results and the previous ones. First, only a small number of patients had HFNC failure in previous studies [10, 16]. Second, the timing of the ROX index is important when interpreting its reliability in predicting HFNC failure [20]. While we considered the 6-h ROX index, previous studies considered the latest value of ROX index within the first four hours [16] or the first 12 h [10] after HFNC initiation. Third, the timing of intubation differed between studies with a median time of two days in our study and of only 10 h in the cohort by Zucman and colleagues [16]. It cannot be ruled out that in our study, although patients were intubated within the first days of ICU admission, these later intubations may not be solely related to the 6-h ROX index.

Our results may suggest that HFNC might be a feasible and valuable first-line ventilatory support in critically-ill patients with COVID-19 with potential benefits such as a lower intubation rate. However, patients should be carefully monitored as there is a significant rate of HFNC failure, especially in the first few hours of management, with the risk of HFNC appearing to decrease after a 48-h HFNO duration. Such strategy deserves to be confirmed by further randomized clinical trials.

We acknowledge some limitations to our study. First, it was a single-center, observational, retrospective study, which implies the possibility of confounding factors. However, the single-center design ensured that the ventilatory management of patients with regard to first-line ventilatory support and intubation criteria was consistent and comparable throughout the study period. In addition, we used a competing risk framework and a propensity score analysis that accounted for changes in standard of care over the study period to strengthen our results. Second, we did not record baseline respiratory parameters before HFNC initiation, but these parameters were not found to be associated with HFNC failure in previous studies [6, 10]. Third, we only assessed a single value of ROX index and it cannot be ruled out that considering a dynamic assessment of ROX index may have been more helpful to identify patients who were more likely to fail. Fourth, the different variants were not systematically screened and therefore we could not assess the potential effect of the different variants on patient ventilatory management and outcomes. Finally, our results are not generalizable to patients who have received immunomodulatory treatments and/or vaccinations and to new emergent SARS-CoV-2 variants.

## Conclusions

In critically-ill COVID-19 patients, while HFNC use as first-line ventilatory support was associated with a lower risk of intubation, more than half of patients had HFNC failure. Risk factors for HFNC failure were SAPS-2 score and extent of CT-Scan abnormalities > 75%. The risk of HFNC failure could not be predicted by the 6-h ROX index but decreased after a 48-h HFNC duration.

## Supplementary Information

**Additional file 1: Table S1.** Patient management and outcomes according to the use of high-flow nasal cannula oxygen. **Table S2.** Patient characteristics, management and outcomes according to the pandemic wave.

## Data Availability

The datasets used and/or analyzed during the current study are available from the corresponding author on reasonable request.

## Abbreviations

COVID-19: Coronavirus disease 19
ICU: Intensive care unit
HFNC: High-flow nasal cannula oxygen therapy
NIV: Non-invasive ventilation
