# Supplementary material for: Use of high-flow nasal cannula oxygen and risk factors for high-flow nasal cannula oxygen failure in critically-ill patients with COVID-19
Source: Respir Res. 2022 Dec 3;23:329. doi: 10.1186/s12931-022-02231-2 (PMC9719644; doi:10.1186/s12931-022-02231-2)
Supplement: Supplementary file 1 — Additional file 1: Table S1. Patient management and outcomes according to the use of high-flow nasal cannula oxygen. Table S2. Patient characteristics, management and outcomes according to the pandemic wave. [file 12931_2022_2231_MOESM1_ESM.docx]

**Use of high-flow nasal cannula oxygen and risk factors for high-flow nasal cannula oxygen failure in critically-ill patients with COVID-19**

Zakaria AIT HAMOU; MD^1,2^ ; Nathan LEVY, MD^1^ ; Julien CHARPENTIER, MD^1^ ; Jean-Paul MIRA, MD PhD^1,2^; Matthieu JAMME MD^3,4^ ; Mathieu JOZWIAK, MD PhD^1,2^

1 : Service de Médecine Intensive Réanimation, Hôpitaux Universitaires Paris Centre, Hôpital Cochin, Assistance Publique – Hôpitaux de Paris, 27 rue du faubourg Saint Jacques, 75014 Paris, France.

2 : Université de Paris, Paris, France

3 : Service de Réanimation médico-chirurgicale, Hôpital Privé de l’Ouest Parisien, Ramsay Generale de Santé, 14 rue Castiglione del Lago, 78190, Trappes, France.

4 : INSERM U1018, Centre de recherche en épidémiologie et santé des populations (CESP), Equipe « Epidemiologie clinique », Université Paris Saclay, 16 avenue Paul Vaillant Couturier, 94800, Villejuif, France

***Corresponding author***

Mathieu JOZWIAK, MD, PhD

Service de Médecine Intensive Réanimation, Hôpitaux Universitaires Paris Centre,

Hôpital Cochin, Assistance Publique – Hôpitaux de Paris,

27 rue du faubourg Saint Jacques, 75014 Paris, France.

[jozwiak.m@chu-nice.fr](mailto:jozwiak.m@chu-nice.fr)

| **Table S1. Patient management and outcomes according to the use of high-flow nasal cannula oxygen therapy.** | | | | | | |
| --- | --- | --- | --- | --- | --- | --- |
| **Variables** | **No HFNC**  **(n=86)** | |  | **HFNC**  **(n=114)** | | **p value** |
|  |  |  |  |  |  |  |
| **Treatments at ICU admission** |  |  |  |  |  |  |
|  |  |  |  |  |  |  |
| Corticosteroids, n (%) | 22 | (25) |  | 94 | (83) | <0.001 |
|  |  |  |  |  |  |  |
| Immunomodulatory treatments, n (%) | 0 | (0) |  | 6 | (5) | 0.01 |
|  |  |  |  |  |  |  |
| Antiviral drugs, n (%) | 28 | (33) |  | 9 | (8) | <0.001 |
|  |  |  |  |  |  |  |
| Low-dose thrombophylaxis, n (%) | 49 | (57) |  | 17 | (15) | <0.001 |
|  |  |  |  |  |  |  |
| Enhanced intermediate-dose thrombophylaxis, n (%) | 22 | (26) |  | 83 | (79) | <0.001 |
|  |  |  |  |  |  |  |
| Curative anticoagulation, n (%) | 9 | (11) |  | 15 | (13) | 1.00 |
|  |  |  |  |  |  |  |
| Antibiotherapy, n (%) | 12 | (14) |  | 58 | (51) | 0.01 |
|  |  |  |  |  |  |  |
| **Ventilatory management** |  |  |  |  |  |  |
|  |  |  |  |  |  |  |
| Non-invasive ventilation, n (%) | 5 | (6) |  | 20 | (18) | 0.02 |
|  |  |  |  |  |  |  |
| Intubation, n (%) | 67 | (78) |  | 78 | (68) | 0.18 |
|  |  |  |  |  |  |  |
| Neuromuscular blocker agents, n (%)* | 67 | (100) |  | 69 | (88) | 0.001 |
|  |  |  |  |  |  |  |
| Prone positioning, n (%)* | 43 | (64) |  | 53 | (68) | 0.37 |
|  |  |  |  |  |  |  |
| Number of prone positioning sessions | 3 | (2-8) |  | 3 | (2-5) | 0.36 |
|  |  |  |  |  |  |  |
| Awake prone positioning, n (%) | 1 | (1) |  | 47 | (41) | <0.001 |
|  |  |  |  |  |  |  |
| Venovenous ECMO, n (%)* | 11 | (16) |  | 3 | (4) | 0.007 |
|  |  |  |  |  |  |  |
| Nitric oxide, n (%)* | 6 | (9) |  | 12 | (15) | 0.66 |
|  |  |  |  |  |  |  |
| **Delays and outcomes** |  |  |  |  |  |  |
|  |  |  |  |  |  |  |
| From onset of symptoms to ICU admission (days) | 7 | (6-10) |  | 8 | (6-11) | 0.53 |
|  |  |  |  |  |  |  |
| From ICU admission to intubation (days) | 0 | (0-0) |  | 2 | (1-4) | <0.001 |
|  |  |  |  |  |  |  |
| Duration of invasive mechanical ventilation (days) | 24 | (13-36) |  | 16 | (7-33) | 0.007 |
|  |  |  |  |  |  |  |
| Tracheostomy, n (%)* | 17 | (25) |  | 13 | (17) | 0.09 |
|  |  |  |  |  |  |  |
| Ventilator-associated pneumonia, n (%)* | 59 | (88) |  | 56 | (72) | 0.02 |
|  |  |  |  |  |  |  |
| Pulmonary embolism, n (%) | 8 | (10) |  | 3 | (2) | 0.06 |
|  |  |  |  |  |  |  |
| Pneumothorax, n (%) | 4 | (5) |  | 4 | (4) | 0.96 |
|  |  |  |  |  |  |  |
| ICU length of stay (days) | 17 | (6-37) |  | 15 | (7-32) | 0.80 |
|  |  |  |  |  |  |  |
| ICU mortality (n,%) | 24 | (28) |  | 31 | (27) | 1.00 |
|  |  |  |  |  |  |  |
| In-hospital mortality, n (%) | 28 | (33) |  | 34 | (30) | 0.79 |
|  |  |  |  |  |  |  |
| n=200. Data are expressed as median (interquartile range) or counts (percentages).  *In patients who were intubated: n=67 in the “No HFNC” group and n=78 in the “HFNC” group.  ECMO: extracorporeal membrane oxygenation; HFNC: high-flow nasal cannula oxygen therapy; ICU: intensive care unit; SAPS: simplified acute physiology score; SOFA: sepsis-related organ failure assessment. | | | | | | |

| **Table S2. Patient characteristics, management and outcomes according to the pandemic wave.** | | | | | | |
| --- | --- | --- | --- | --- | --- | --- |
| **Variables** | **First wave**  **(n=82)** | |  | **Second wave**  **(n=118)** | | **p value** |
|  |  |  |  |  |  |  |
| **Clinical characteristics** |  |  |  |  |  |  |
|  |  |  |  |  |  |  |
| Age (years) | 61 | (51-69) |  | 68 | (59-74) | 0.003 |
|  |  |  |  |  |  |  |
| SAPS2 | 64 | (40-77) |  | 41 | (31-60) | <0.001 |
|  |  |  |  |  |  |  |
| SOFA score on ICU admission | 10 | (5-12) |  | 5 | (3-7) | <0.001 |
|  |  |  |  |  |  |  |
| Male gender, n (%) | 57 | (70) |  | 84 | (71) | 0.92 |
|  |  |  |  |  |  |  |
| Body mass index (kg/m²) | 27 | (25-30) |  | 28 | (26-32) | 0.27 |
|  |  |  |  |  |  |  |
| Obesity, n(%) | 22 | (27) |  | 38 | (32) | 0.51 |
|  |  |  |  |  |  |  |
| Arterial hypertension, n (%) | 33 | (40) |  | 76 | (65) | 0.001 |
|  |  |  |  |  |  |  |
| Diabetes mellitus, n (%) | 23 | (28) |  | 41 | (35) | 0.40 |
|  |  |  |  |  |  |  |
| Coronary artery disease, n (%) | 8 | (10) |  | 20 | (17) | 0.21 |
|  |  |  |  |  |  |  |
| Stroke, n (%) | 5 | (6) |  | 7 | (6) | 1.00 |
|  |  |  |  |  |  |  |
| Chronic respiratory disease, n (%) | 10 | (12) |  | 21 | (18) | 0.38 |
|  |  |  |  |  |  |  |
| Chronic kidney disease, n (%) | 7 | (8) |  | 20 | (17) | 0.19 |
|  |  |  |  |  |  |  |
| Cirrhosis, n (%) | 1 | (1) |  | 2 | (2) | 1.00 |
|  |  |  |  |  |  |  |
| Neoplasia, n (%) | 12 | (15) |  | 26 | (22) | 0.26 |
|  |  |  |  |  |  |  |
| Blood type, n (%)* |  |  |  |  |  | 0.72 |
| A | 32 | (41) |  | 48 | (42) |  |
| B | 9 | (11) |  | 19 | (16) |  |
| AB | 5 | (6) |  | 7 | (6) |  |
| O | 33 | (42) |  | 41 | (36) |  |
|  |  |  |  |  |  |  |
| Nosocomial COVID-19, n (%) | 1 | (1) |  | 15 | (13) | 0.007 |
|  |  |  |  |  |  |  |
| CT-Scan, n (%) | 67 | (82) |  | 103 | (87) | 0.38 |
|  |  |  |  |  |  |  |
| CT-Scan abnormalities (%) |  |  |  |  |  | 0.06 |
| <10% | 3 | (4) |  | 2 | (2) |  |
| 10-25% | 14 | (21) |  | 26 | (25) |  |
| 25-50% | 22 | (33) |  | 38 | (37) |  |
| 50-75% | 20 | (30) |  | 33 | (32) |  |
| >75% | 8 | (12) |  | 4 | (4) |  |
|  |  |  |  |  |  |  |
| Norepinephrine, n (%) | 55 | (67) |  | 67 | (57) | 0.19 |
|  |  |  |  |  |  |  |
| Renal replacement therapy, n (%) | 17 | (21) |  | 27 | (23) | 0.85 |
|  |  |  |  |  |  |  |
| **Biological variables at ICU admission** |  |  |  |  |  |  |
|  |  |  |  |  |  |  |
| Lymphocytes (x10^9^/L) | 0.75 | (0.54-1.11) |  | 0.74 | (0.48-1.12) | 0.75 |
|  |  |  |  |  |  |  |
| Fibrinogen (g/L) | 5.9 | (5.1-7.2) |  | 5.9 | (5.1-7.0) | 0.94 |
|  |  |  |  |  |  |  |
| D-Dimers (μg/L) | 2183 | (1193-5568) |  | 1001 | (636-1909) | <0.001 |
|  |  |  |  |  |  |  |
| Protein C reactive (mg/L) | 156 | (112-245) |  | 120 | (69-190) | 0.003 |
|  |  |  |  |  |  |  |
| Procalcitonin (ng/L) | 0.56 | (0.20-1.50) |  | 0.29 | (0.13-0.73) | 0.03 |
|  |  |  |  |  |  |  |
| Ferritin (ng/mL) | 1051 | (673-2770) |  | 1065 | (581-1899) | 0.87 |
|  |  |  |  |  |  |  |
| Interleukin-6 (pg/mL) | 162 | (82-349) |  | 61 | (22-203) | 0.009 |
|  |  |  |  |  |  |  |
| Troponin (ng/L) | 15 | (11-81) |  | 17 | (10-43) | 0.50 |
|  |  |  |  |  |  |  |
| **Treatments at ICU admission** |  |  |  |  |  |  |
|  |  |  |  |  |  |  |
| Corticosteroids, n (%) | 2 | (2) |  | 114 | (97) | <0.001 |
|  |  |  |  |  |  |  |
| Immunomodulatory treatments, n (%) | 6 | (7) |  | 0 | (0) | 0.01 |
|  |  |  |  |  |  |  |
| Antiviral drugs, n (%) | 36 | (44) |  | 1 | (1) | <0.001 |
|  |  |  |  |  |  |  |
| Low-dose thrombophylaxis, n (%) | 59 | (72) |  | 7 | (6) | <0.001 |
|  |  |  |  |  |  |  |
| Enhanced intermediate-dose thrombophylaxis, n (%) | 12 | (15) |  | 93 | (79) | <0.001 |
|  |  |  |  |  |  |  |
| Curative anticoagulation, n (%) | 9 | (11) |  | 18 | (15) | 0.51 |
|  |  |  |  |  |  |  |
| Antibiotherapy, n (%) | 62 | (76) |  | 56 | (47) | <0.001 |
|  |  |  |  |  |  |  |
| **Ventilatory management** |  |  |  |  |  |  |
|  |  |  |  |  |  |  |
| HNFC, n (%) | 19 | (23) |  | 95 | (80) | <0.001 |
|  |  |  |  |  |  |  |
| Non-invasive ventilation, n (%) | 0 | (0) |  | 25 | (21) | <0.001 |
|  |  |  |  |  |  |  |
| Intubation, n (%) | 70 | (85) |  | 75 | (63) | 0.001 |
|  |  |  |  |  |  |  |
| Neuromuscular blocker agents, n (%)** | 67 | (96) |  | 69 | (92) | 0.49 |
|  |  |  |  |  |  |  |
| Prone positioning, n (%)** | 43 | (61) |  | 53 | (71) | 0.29 |
|  |  |  |  |  |  |  |
| Number of prone positioning sessions | 2 | (0-4) |  | 1 | (0-4) | 0.45 |
|  |  |  |  |  |  |  |
| Awake prone positioning, n (%) | 4 | (5) |  | 44 | (37) | <0.001 |
|  |  |  |  |  |  |  |
| Venovenous ECMO, n (%)** | 11 | (16) |  | 3 | (4) | 0.02 |
|  |  |  |  |  |  |  |
| Nitric oxide, n (%)** | 6 | (8) |  | 12 | (16) | 0.21 |
|  |  |  |  |  |  |  |
| **Delays and outcomes** |  |  |  |  |  |  |
|  |  |  |  |  |  |  |
| From onset of symptoms to ICU admission (days) | 11 | (6-10) |  | 7 | (5-10) | 0.61 |
|  |  |  |  |  |  |  |
| From onset of symptoms to HFNC initiation (days) | 10 | (7-12) |  | 8 | (5-11) | 0.10 |
|  |  |  |  |  |  |  |
| From ICU admission to HFNC initiation (days) | 0 | (0-0) |  | 0 | (0-0) | 0.45 |
|  |  |  |  |  |  |  |
| From ICU admission to intubation (days) | 0 | (0-0) |  | 2 | (0-4) | <0.001 |
|  |  |  |  |  |  |  |
| Duration of HFNC (days) | 0 | (0-1) |  | 3 | (1-6) | <0.001 |
|  |  |  |  |  |  |  |
| Duration of invasive mechanical ventilation (days) | 23 | (9-39) |  | 6 | (0-16) | <0.001 |
|  |  |  |  |  |  |  |
| Tracheostomy, n (%)** | 17 | (24) |  | 13 | (17) | 0.31 |
|  |  |  |  |  |  |  |
| Ventilator-associated pneumonia, n (%)** | 59 | (84) |  | 56 | (75) | 0.22 |
|  |  |  |  |  |  |  |
| Pulmonary embolism, n (%) | 8 | (10) |  | 3 | (2) | 0.06 |
|  |  |  |  |  |  |  |
| Pneumothorax, n (%) | 4 | (6) |  | 4 | (5) | 0.92 |
|  |  |  |  |  |  |  |
| ICU length of stay (days) | 25 | (12-43) |  | 12 | (5-25) | <0.001 |
|  |  |  |  |  |  |  |
| ICU mortality (n,%) | 22 | (27) |  | 33 | (28) | 0.99 |
|  |  |  |  |  |  |  |
| In-hospital mortality, n (%) | 24 | (29) |  | 38 | (32) | 0.77 |
|  |  |  |  |  |  |  |
| n=200. Data are expressed as median (interquartile range) or counts (percentages).  *Data are available for 194 patients : 79 patients in the first wave and 115 patients in the second wave.  **In patients who were intubated : n=70 in the first wave and n=75 in the second wave.  ECMO: extracorporeal membrane oxygenation; HFNC: high-flow nasal cannula oxygen therapy; ICU: intensive care unit; SAPS: simplified acute physiology score; SOFA: sepsis-related organ failure assessment. | | | | | | |
